# Remarks on the Gibraltar Epidemic

**Published:** 1831-06

**Authors:** D. Barry


THE LONDON

### Medical and Physical Journal.

No. 388, VOL. LXVI.] JUNE 1831. [No. 60, *New Series.*

"For many fortunate discoveries in medicine, and for the detection of numerous errors, the world is indebted to the rapid circulation of Monthly Journals; and there never existed any work to which the Faculty, in Europe and America, were under deeper obligations than to the 'Medical and Physical Journal of London,' now forming a long but invaluable series."—RUSH.

### ORIGINAL PAPERS, AND CASES,

### OBTAINED FROM PUBLIC INSTITUTIONS AND OTHER AUTHENTIC SOURCES.

### GIBRALTAR EPIDEMIC.

*Remarks on the Gibraltar Epidemic.* By D. BARRY, M.D.

From p. 417.

*To the Editor of the London Medical and Physical Journal.*

SIR: I have carefully read over the controversial observations connected with the late Gibraltar fever, given in your last three numbers, and am sorry to observe that they shed but little, if any, light on the history of that disease. Vague declamatory assertions, loosely mixed up with irrelevant personalities, whatever attempts may be afterwards made to palliate their want of authenticity, cannot add to our useful knowledge: precise notions are not to be thus acquired. Facts alone, and these placed beyond the reach of sophistry, ought to constitute the basis of just conclusions. Theories change and are forgotten, but facts remain for ever.

The interest of your readers, sir, and consideration for my own dignity, demand that, in noticing the observations alluded to, I should not descend to meet their author with the uncourteous weapons which he has chosen, nor follow him through the narrow path which he still thinks proper to pursue. The epigraph of your Journal forbids such a course: "you are forming a long series;" and it shall be my endeavour to render that series, so far at least as the Gibraltar fever is concerned, as valuable and as authentic as the extent of my information will admit.

In my present communication, I should feel but too happy could I, with propriety, omit every thing purely personal to

Reel 181-36-5 (pp. 475-94, contin.)

myself, and at once direct my undivided attention to the main points at issue; but the letters which you have recently published, render the adoption of such a plan impossible. I had, indeed, at one time, determined to pass over the calumnious incivilities of all my opponents in silent scorn, and should now do so, were it not for the apprehension, that my leaving them unnoticed might be construed into an admission of my inability to refute them. I am anxious, in short, that neither myself nor my friends should be thought to feel the

> "Pudet hæc opprobria nobis,
> Et dici potuisse, et non potuisse refelli."

My plan of proceeding shall be this: The ungracious task, just alluded to, must occupy the first part of my letter. That dismissed with all due brevity, I shall address myself, in the most direct manner, to the two following questions, viz.

1. Were the germs of the late Gibraltar epidemic fever imported into that territory, during the summer or autumn of 1828, and afterwards propagated there?

2. Was that fever, wherever it may have originated, contagious in Gibraltar, under any circumstances?*

With the hope of enabling all who take an interest in these questions to judge for themselves, and to arrive at something like a satisfactory solution of them, I shall attempt to place, in chronological array, brief authentic details of the early and more important events of the epidemic, and shall then give a general statistic view of the recorded opinions of those who witnessed the events, and who, from education and habits, were best fitted to draw right deductions.

Should the correctness of any of my details be questioned, or any of the documents on which they repose be considered apocryphal, let them be temperately and fairly tested; but let me entreat you, Mr. Editor, not to persist in admitting into your pages useless invective, in the discussion of questions, which ascertained facts, and authentic, unprejudiced records alone, can illustrate.

1. The sectarians of the Chervin school have, as you know, long insinuated, and, emboldened perhaps by my silence hitherto, now openly assert, that, upon my first appearance in

* "Contagium verò est similis affectionis morbosæ in alio corpore, per inquinamentum a morboso corpore communicatum, productio.

"Ad contagium verò requiruntur tria. Ipsum corpus contagiosum quod alia inficit; affectio præter naturam quæ alteri communicatur, et corpus quod inficitur.

"Vehiculum duplex est aër et fomes aliquis.

"Fomitis rationem habet quidquid contagii semina in se recipere et alio transferre potest."—SENERTUS, *anno* 1655.

Gibraltar, in 1828, that is, from the 10th up to "eight or ten days after the 25th of November," I warmly advocated the negative, but that, shortly after the arrival of Dr. Pym, I suddenly made a "*volte face*," and espoused the affirmative of both the questions already stated. Mark the dates: I arrived on the 10th, Dr. Chervin on the 23d, Dr. Pym on the 29th November. A host of that sort of anile, gossiping evidence, in which Mr. Fraser and his friends so largely deal, is adduced to shew that, about the same time, I wrote a memoir, or letter, in which, it was said, I argued, at great length, that the prevailing disease had its origin on the rock, and was by no means contagious. These vague reports, it now appears, constitute the "corpus delicti" of the mysterious impeachments and disclosures which Mr. Fraser and his prompters have held suspended over my head, during the last eight months.

Fortunately, however, for the consistency of my opinions on the subjects of contagion, and the importation of disease generally, they were published to the world, three years before I had the pleasure of knowing either Dr. Pym or Dr. Chervin; for I met both these gentlemen, for the first time, in Gibraltar, during the late epidemic. Now, sir, as you have honoured me so highly as to entertain the hearsay comments of my adversaries, not only on the tenor, but even on the pretended motives and history of my opinions, I may be permitted to hope that you will also afford room to my own account of their rise and developement.

Nearly seven years ago, I exhibited, as you are well aware, two series of physiological experiments in Paris, before separate commissions, appointed, *ad hoc*, by the Institute and Royal Academy of Medicine. One series related to the function of absorption, and was directed, amongst other objects, to determine the absolute and relative absorbing powers of the different tissues and organs, of which man, and the higher classes of animals, are composed. The details of these experiments, with my conclusions, and the reports of the two commissions, were published by me in Paris, in 1825, and afterwards in this metropolis, in 1826, under the title of "Experimental Researches."*

The strong bearing which the following experiment, and the remarks attached to it at the time, seem to have on the

* "Experimental Researches, on the Influence exercised by Atmospheric Pressure on the Progression of the Blood in the Veins, by David Barry, m.d." London: Underwood, 1826.—This work, long out of print, may be found in the military libraries at Chatham and Gibraltar.

questions at issue, will, I trust, plead my apology with your readers for quoting from my own work.

"*Twenty-fourth experiment.** One grain of alcoholic extract of nux vomica, dissolved in two ounces of distilled water, and injected through the trachea, into the lungs of a dog, produced tetanic spasm of the limbs and opisthotonos, within the tenth second from the commencement of the injection; and death in less than two minutes.† He breathed freely after the syringe was removed.‡

"A similar quantity of the same liquid was injected through a stop-cock, which had been previously fitted into the trachea of another dog, and the stopper was turned the moment the injection was completed. The symptoms came on some seconds later. Opening the stop-cock, and allowing the animal to breathe, did not prolong his existence.

"These experiments account for the communication of disease without contact. The infective matter of smallpox is more abundantly and more fatally taken into the system, by breathing the atmosphere of the variolous, than by inoculation; the plague, by inhaling the effluvia of the pesthouse. In short, whatever poison is capable of being suspended, or dissolved in the air as a menstruum, must inevitably pass into the blood of those who respire this air, thus infected. "Qui cum non respirare non possunt, contagium miseri, evadere nequeunt." (GALEN, 5, 96, G.)

§ "If the air around an infected individual, or bale of goods, could be so impregnated with the emanation of variolous or plague virus, *or with the germs of any other disease whatever*, as the distilled water was with nux vomica, in the twenty-fourth and twenty-fifth experiments, there cannot exist even the shadow of a doubt, that a sound individual, respiring that air, would be more rapidly, and more abundantly poisoned than he could be by inoculation.

"If one infected individual cannot furnish enough of virus to charge the atmosphere around him with the seeds of his disease, we know that a greater number can; and if the air be not disposed at one time to hold these germs suspended, we know that at other times it is so disposed. Therefore, whilst men have lungs constructed as these organs are at present; whilst the mucous surface of these lungs is exposed to the contact of every thing the atmosphere holds in solution; and whilst it is certain that the most fatal poisons may be thus deposited on the most rapidly absorbing

* Vide opus cit. p. 139 et seq.

† The Strychnos Nux vomica, which owes its energy, as a poison, to the strychnia it contains, would, in this very minute dose, produce no effect applied to the external cellular tissue, and but trifling and slow effects in contact with the mucous surface of the stomach of the very animal, which it thus almost instantaneously destroyed.—D. B.

‡ This experiment I repeated, on several occasions, at my public lectures, in London.—D. B.

§ Op. cit. p. 144.

tissue of the whole frame, the healthy should be carefully, and distantly, separated from the infected; nor should the former ever, under any circumstances, respire the air which the emanations from the latter may have poisoned.

"From what has been said on the subject of specific morbid poisons, may be seen the incorrectness, nay, even the dangerous tendency of the distinction lately attempted to be established by some writers, between *contagion and infection.*

"If contagion be considered as having reference only to the necessity of contact between any of the specific poisons and an absorbing surface, then all the diseases communicated by morbific matter, whether solid or gaseous, must be ranged under the head of contagious. But if it refer to the presumed necessity of contact between sound and infected individuals, then none of the diseases alluded to can be called contagious, because this kind of contact is in no case necessary to their being communicated.

"The word infect, and its derivatives, clearly convey the idea of something noxious introduced into the system: they admit of no quibbling ambiguity, and should, in sanitary logic, universally supersede the use of the word contagion, and its adjectives."*

By the above extracts, you will perceive that, nearly four years before I knew Dr. Pym, except from reputation, (for, as I have already stated, we met for the first time in Gibraltar in November 1828,) I had fully made up my mind on the subject of contagion generally, or rather infection, as I wished to have it called. I had arrived, in fact, by the road of inductive experiment, to the conclusion, that emanations from a diseased, applied to the pulmonary mucous tissue of a sound, individual, may become, under proper circumstances, a direct poison.

Full of the impressions which these experiments had left on my mind, and proud of their having been verified, and honourably quoted, both in Europe and in America, I was selected by the Medical Board to proceed to Gibraltar, during the late epidemic. I confess that I undertook the mission with reluctance, not being at all ambitious to put to the test, upon my own lungs, the speculations I had so often realised upon those of dogs and rabbits. I landed, however, and pitched my tent on the Neutral Ground, on the 10th November, and on the 15th commenced the annexed letter, which, as you will perceive by the letter itself, I was not able to com-

* "Observe how, in a little note, subsequently added for the emergency, to that letter, (viz. of 14th November, 1828,) he makes a desperate effort, by telling us that he uses the word *infectious,* as synonymous with contagious, to explain away what he had said about the accumulation of infectious disease." (See Mr. Fraser's letter, in your May number, p. 401.)—D. B.

plete and give in before the 21st, having been attacked by the disease in the interim.*

This is the very "Memoir" which the Chervinists do me the honour to claim as having been written in support of their creed; this is the paper, to the contents of which I am accused of having myself become an apostate, in order to secure the patronage of Dr. Pym.† This is the overwhelming dis-

* Mr. Fraser, in his last letter, adduces, as a proof of the non-contagious nature of the Gibraltar fever, his own assertion, that several medical gentlemen, amongst others, Mr. Burkitt, assistant surgeon of the 94th regiment, escaped infection, though in constant attendance upon the sick, towards the close of the epidemic. "*Credite posteri!*" Mr. Burkitt was dead before the epidemic began; he died in July, as may be seen by the Army List for September of that year. What must your readers now think as to the precision of facts drawn from the stores of such a memory; of arguments concocted by a mind capable of resting conclusions upon such blundering data? "But more of this hereafter."

As to my own case: vomiting, with gastric pains and diarrhœa, having been amongst the earliest symptoms, I caught at the hope which my medical friends allowed me to cherish, for the first twenty-four hours, that it might be only an attack of ordinary cholera, and dictated from my sick bed, on the 18th November, the words quoted by Mr. Fraser. This I did with a view to pass the disease, whatever it might be, in the pure air of the Neutral Ground, and to save myself from an overweening sanitary caution, which would otherwise have consigned me, as it had many others, to the vicinity of the Naval Hospital in the south, the path to which, like that leading to the lion's den in the fable, was crowded with footsteps pointing inwards.

The following document will show how far I was justified in writing to Dr. Broadfoot, on the 18th January, 1829, a private, playful note, containing these words: "Don't forget to mention, when you write to your friends, that I have had an attack of the fever."

"A true copy," (Signed) "R. Tucker."

(copy.)

"*Gibraltar; 1st January*, 1829.

"I saw Dr. Barry several times during his attack, and have no doubt that, though not very severe, yet that his was a real case of the prevalent epidemic, which I expressed to him at the close of his illness.

(Signed) "ALEXANDER BROADFOOT,
"Inspector of Health, and Deputy
Inspector of Hospitals."

A true copy, D. Barry.

† This distinguished individual, now Sir William Pym, acted as principal medical officer in Gibraltar, from the date of his landing to the close of the epidemic, and, as such, had an opportunity of recommending various officers of his department for promotion. Of some of these recommendations it may be observed, that, if serving those who opposed his views be a proof of impartiality, he cannot be accused of interested motives. For my own part, I went out to Gibraltar with the local rank, and a promise in my pocket, of the effective step, physician to the forces, which I afterwards obtained: nor do I consider myself at all indebted to Sir William Pym for the fulfilment of that promise, which would have taken place, even though I never had the pleasure of knowing him. However highly I may value Sir William's private friendship, truth compels me to say, that I owe nothing to his patronage.—D. B.

closure, with which I have been so long and so pompously threatened by these gentlemen, and to support which they now bring up their whole strength. "Parturiunt montes!"

The civil and military secretaries are supposed to have seen the Memoir; the captain of the port glanced a critical eye over it, but is not quite sure whether he may "not have been mistaken" as to its purport; the surgeon of the Civil Hospital heard a few sentences of it read, on the subject of localities, and thought them quite correct. Then come the ladies; I had almost said, the old women. M. Chervin writes from Paris, to his "dear Mr. Fraser," that Miss Broadfoot, the near relative of the principal medical officer of Gibraltar, to whom the Memoir was addressed, told him that she had actually looked into the paper, and that it contained fifty or sixty pages; but, by counting them, you will perceive, that this fair élève is, most ungallantly, made to see double: Dr. Broadfoot could not find time to read the Memoir himself, though he appears, by the endorsements, to have become acquainted with its purport; no doubt, through Miss Broadfoot.

Look back at this evidence, I pray you, after you have read the Memoir, and see how conclusive it is as to the contents; observe the amiable and manly zeal with which Mr. Fraser and his prompters strive to justify their libellous assertions, after having been dared to the proof. The very play-hours of life are not sacred to them. The social conversation of the dinner table and the drawing room; the unsuspecting prattle of an interesting and accomplished young lady; the welcome of a confrère, are all registered and published: neither sex nor circumstance, it would appear, can save from publicity whoever may have had the misfortune to exchange a syllable with M. Chervin on his eternal day-dream, "la fièvre jaune." The friendly advice of a senior in rank and years, dictated by compassion for the errors and obstinacy of a junior, is perverted, by the very man whom it was intended to serve, into an attempt at corrupt intimidation. In such a circle, let me ever be the accused.

The subjoined note from M. Trousseau to M. Louis will shew what a safe companion M. Chervin must be;* and how correctly he must have understood my sentiments on contagion, when he registered as the ground of a *document*, or an

* "But it (M. Chervin's letter) will shew further, and on the evidence of one (viz. M. Trousseau,) whose veracity I do not suppose Dr. Barry will be inclined to question, that this advocate of what I consider a very bad cause has woven a web for himself, from which it will not be very easy for him to emerge." (Fraser's letter, March number, p. 207.)—D. B.

accusation against me, the very conversation in which, as a British officer, I bade him welcome to a British fortress.* But to return.

I send you the original Memoir in my own handwriting, bearing the following endorsement written by Mr. Tucker, chief clerk of the medical department, on one side, by order of Dr. Broadfoot.

"1828; Gibraltar, 15 November. Dr. Barry, physician to the forces, his views of the disease; means of prevention, localities, &c. &c.; for submission to his Excellency."

On the other side of the cover, as it is folded, is another endorsement by myself; thus:

"Not submitted to his Excellency the Lieutenant Governor, but suppressed by Dr. Broadfoot, and returned to me, after eight or ten days, with the observation, that, as the views it contained were

* "À Mons. le Docteur Louis, Rue Bergêre, à Paris.

"Voici, mon cher camarade, ce qu'en mon âme et conscience je me rappele des entretiens de Barry et de Chervin. Une fois sans plus, j'ai entendu Barry dire à Chervin, c'était quatre ou cinq jours après notre arrivé à Gibraltar, qu'il était bien aise qu'il eut été envoyé par le gouvernement François, et que sans doute il se convaincrait, comme lui (Barry) avait eu déjà l'occasion de la faire, que la fièvre de Gibraltar se propageoit par infection. Je suis bien aise, lui dit Chervin, que vous ayez sur ce point, les mêmes idées que moi. Barry le lui confirme de nouveau, et la conversation se mit aussitôt sur un autre chapitre.

"À quelques jours de la, je rencontrai Barry aupres de Bella vista, à l'Europe. Après la salutation d'usage, Eh, bien, lui dis-je, vous êtes donc infectioniste; voila Chervin bien heureux! Oui, me dit-il, je le suis. Ah, ça, repliquoi-je, comment m'entendez vous? Pensez-vous que si deux cents malades de cet hôpital, et je lui designais l'Hôpital de la Marine, étaient enlevés en ballon avec quelques individus bien portants, ces derniers ne contracteraient pas la maladie? Si certainement, répondit-il, avec vivacité, mais ce serait par infection de l'air, comme on contracte la rougeole, la petite verole, sauf inoculation, lorsqu'on se trouve dans une localité ou l'air a été infecté par des personnes attéintes de ces maladies. Eh! parbleu, mon cher confrère, lui dis-je, en riant, vous êtes loin de compte avec Chervin, et desormais vous êtes son ennemis; car votre infection est de la contagion. Vous êtes contagioniste, et archicontagioniste.

"En effet, Barry entendait le mot infection dans le sens ou l'entendent encore, beaucoup de médecins, qui ne regardent comme maladies contagieuses qui celles qui se transmettent par un contact immediat.

"Voici ce que j'en sais. J'affirme sur l'honneur que je n'ai assisté qu'à une seule conversation entre Chervin et Barry, dans laquelle ce dernier ait dit qu'il était infectioniste, et encore cette conversation se borna-t-elle à deux ou trois mots de compliments sans aucun detail.

Addio, tout à vous de cœur,

(Signé) "A. TROUSSEAU."

"*Paris;* 15 *Mars,* 1831."

so very similar to those he intended to submit himself to his Excellency, he had not submitted it.

(Signed) "D. BARRY, Staff Surgeon."

"*Gibraltar; 2d August*, 1829."

There is also, on the back of this original paper, a certificate from Mr. Tucker, now deputy purveyor, as to the authenticity of the paper, and as to his own handwriting in the first endorsement, in these words:

"The above endorsement is in my handwriting, and having inspected the paper this day, I have no hesitation in declaring that I am convinced it is the same which came into my hands at Gibraltar, as chief clerk of the medical office, to be endorsed, and returned to the principal medical officer, as above dated.

(Signed) "R. TUCKER, Deputy Purveyor."

"*London; February 3d*, 1831."

But lest all this should not be enough to satisfy the very liberal, anticipating doubts of Mr. Fraser and his friends, I send you the very copy which that highly-gifted young surgeon, Mr. Moore, was kind enough to draw up for me in Gibraltar, from the original rough sketch, and which M. Chervin mentions in his letter to Mr. Fraser already quoted. This is the very copy which I submitted to his Excellency Sir George Don, through his military secretary, Colonel Marshall, when I found that the original had been so unhandsomely kept back by Dr. Broadfoot.* May I beg that you will print from this copy; it contains no interpolations, and the high-principled young man, who penned it, will be proud to acknowledge his own handwriting. There is yet another copy of this paper, which I cannot send, but to which I can refer you and your readers: it was forwarded by me from Gibraltar to Sir James M'Grigor, on the 27th November, 1828, and may be seen at the Medical-board office (where it has lain ever since), on application to Dr. Gordon, to whom our science is largely indebted for the mine of authentic information which he has, with much labour, collected and arranged.

These details will serve as tests, by which to estimate the

"*Europa; 2d December*, 1828.

* "My dear sir: Sir George has read, with much interest, your Memoir on the subject of the fever, herewith returned, and requests you will lay it before Dr. Pym. Hereafter, he hopes the public will have the benefit of your thoughts on this subject, more in detail.

"Believe me, my dear sir, very truly yours,

"J. MARSHALL."

"To Dr. Barry."

reasoning powers of M. Chervin and his followers, as well as the probable value of the evidence which men, who can build conclusions on such data, may be supposed capable of bringing forward. I assume no merit to myself for having permitted these gentlemen to commit themselves by publishing to the world, as facts, and by inducing writers, otherwise respectable, to repeat as authentic, such childish gossip. I could not even guess at the nature of the threatened disclosures, and certainly did not anticipate that my medical opinions would be impeached on the evidence of a young lady. But it is quite plain that this playful élève, observing the credulous enthusiasm with which the peripatetic Doctor was amassing *documens*, archly resolved to have a laugh at his expense, by pursuading him that my letter to her relative contained twice the number of pages it really did, and was entirely on his side of the question.

There is but one word more which I would prefix to the letter, and it is this: that, anxious as I was to put my opinions early on record, lest I might be thought to have borrowed them from any of the parties already formed before my arrival, I was not able to bring my mind to the task wholly umbiassed, particularly as to the pretended density of the population, as will be seen by the Memoir. Some persons, who (as I do not mean to betray private conversations) shall be nameless, took great pains to impress on my mind, that the number of inhabitants in the town of Gibraltar alone, at the breaking out of the epidemic, could not have been less than thirty thousand, besides the military; and as these persons were of the highest respectability, and ought to have had access to the most authentic sources of information, I found it difficult to doubt their assertions. But here is the Memoir itself.

"To Alexander Broadfoot, m.d. Inspector of Hospitals, p.m.o. &c.

"*Neutral Ground, Gibraltar; 15th November*, 1828.

"Sir: The mission on which I have had the honour of being sent to this fortress from London, by the government at home, having for its especial object to assist, by every means in my power, in arresting the spread and fatality of the very destructive disease now committing such ravages amongst the inhabitants and the troops, but particularly amongst the latter, I feel it to be my duty to take the very earliest opportunity of submitting, through you, as principal medical officer, to his Excellency Sir George Don, such suggestions on this subject as my observation and inquiries, since I landed, have pressed on my mind.

"I shall not, for the present, enter into any disquisition on the much agitated questions connected with contagion, the precise nature and pathology of the disease, &c.* The simple and melancholy facts, that this disease is highly destructive of human life, and that it spreads, (as remarked by his Excellency, when I had the honour of being presented to him,) furnish quite ground enough, to men of common sense, on which to institute the most immediate, and the most energetic measures to arrest its progress, or, at least, to diminish the frightful mortality which it continues to produce.

"These are the primary objects to be aimed at; and to the speedy attainment of these, all the plans that have already, or that may hereafter be adopted, ought to tend.

"Previously to proposing any sanitary arrangements, I shall beg leave to lay before you my own unbiassed views as to the causes that have hitherto kept up, and still, in my opinion, assist in the propagation and the fatality of the present epidemic.

"1st. As to the localities and exposure of the town, and some of the military buildings, of Gibraltar.

"The rock, I conceive, may be looked upon as an immense wall, running nearly north and south, for about two miles. When the wind blows directly, or at a large angle, against the eastern face of this wall, there must be a column of comparatively, if not absolutely, stagnant air, resting on the western face of the rock, as its base, and terminating at those points where it (the rock) ceases to afford shelter from the easterly winds. In the most unagitated part of this stagnant atmosphere the town of Gibraltar is built.

"When the wind blows directly against the western face of the rock, this stagnant column of air is not dispersed: it is only forced in upon the inhabited parts of the mountain.

"Under these circumstances, particularly during the easterly winds, it is not to be wondered at that stench, and an oppressive atmosphere are complained of in many of the buildings, civil as well as military, because the gases that produce the stench are neither dispersed nor sufficiently diluted.

"Constant agitation, and change of the air we breathe, are amongst the most indispensable conditions necessary to render it healthful. These conditions have been wanting this summer and autumn, in Gibraltar. During the former season, westerly winds, I am told, prevailed; during the latter, easterly winds have been constant, until within a few days ago.

"Even under these disadvantageous circumstances, a moderate number of people might continue to enjoy an ordinary state of public health; more particularly, if favored, as the inhabitants of Gibraltar have been for some years, by civic improvements, and well directed attention to the state of the sewers and drains. But, if the number of people be increased, so as to render them almost in-

* "As my proof, I beg to offer them the following letter from Dr. Chervin. This letter will, I think, shew that I have not made a vague statement respecting that document." (See M. Chervin's letter to Mr. Fraser, Med. and Phys. Journal, March 1831, p. 207.)—D. B.

credibly disproportionate to the space they inhabit, disease of some kind must soon thin them down to the level of the supply of atmosphere on which they have to exist.

" I shall not go so far as to assert, that the state of things which I have detailed has produced the present epidemic; but this I will say, without fear of contradiction, that wherever it originated, its fatality and its dissemination have been largely promoted by undue accumulation of human beings, whether sick or healthy, in an unagitated atmosphere.

" The sanitary measures which this view suggests, are evident:

" 1st. A dispersion of the inhabitants and troops, as far without the boundaries of the stagnant column of air, already alluded to, as the actual circumstances of the fortress and its dependencies will admit.

" 2d. The avoiding accumulation, even beyond the bounds of the stagnant column, either of the healthy or of the sick, but particularly of the latter.

" The whole of the bay and Neutral Ground I conceive to be, for some feet above their surfaces, beyond the limits of the unagitated atmosphere, because there must be, at all times, whenever there is any wind, a current of air passing from one sea to the other.

These situations have continued to enjoy a comparative exemption from the reigning epidemic, for those who have abstained from living much in the focus of disease and accumulation, in the various depressions on the western face of the rock.

" The scattering of the troops and inhabitants over the Neutral Ground, and amongst the shipping, so far as it has been carried into effect, has been a most salutary measure; but the establishment of hospitals within the town, and in the unventilated ravines of the western side of the mountain, has been, and still is, the principal cause to which the frightful mortality, now going on, is attributable.

" The Naval Hospital affords a most striking illustration of the truth of this assertion. One in two of the sick of some regiments treated there has perished, and the unexampled number of eight orderlies and two hospital sergeants of another corps were fatally attacked, whilst attending their regimental sick. In short, this hospital which, in ordinary times, is admirably well calculated for the purposes for which it was originally intended, has been, from the beginning, and is now, for its extent, the most fertile source that I have yet been made acquainted with, of infection and death in the present epidemic.

" In framing and carrying into execution all measures intended to arrest the spread and fatality of the present fever, I would set out with these two leading principles, viz.

" 1st. That the central and most densely inhabited district of the Rock has been, and still is, the chief source and focus of infection.

" 2d. That every accumulation of sick, particularly within the stagnant column of air already alluded to, immediately becomes a fresh source of infection and death, as is proved by the military (Naval) hospital, as well as by the civic establishments of this kind within the town.

"Let the sick, then, of each regiment be treated regimentally in hospitals attached to their respective camps, placed on as large a space as possible, and well exposed to free ventilation. Let arrangements be made to save the healthy soldier, who has never been affected by the epidemic, from doing duty, for the present, of any kind, within the works, or near the accumulated sick. Let it be laid down as a principle, that, by avoiding a certain degree of accumulation of morbid effluvia, whether from dead or living matter, by seeking free ventilation, and observing the most scrupulous cleanliness, the liability to infection will be diminished, at least, a hundred-fold. By acting upon this simple view of the subject, much valuable time will be saved, minute quarantine arrangements will become of less importance. They are generally inefficient, because they cannot be even ordered amongst military men, until the mischief is done, if any there be, and are in most cases absolutely impracticable, from local circumstances, but more particularly at present, from the very limited extent of the territory of Gibraltar.

"If it be proved that a previous attack of this disease affords even a comparative exemption from a second, let the men who have passed through it be returned immediately into town, and let them be employed in those duties that are indispensable to the safety of the fortress. Let the remainder of the troops be scattered over the Bay and Neutral Ground, and the more exposed situations of the south, as widely as the extent of the territories of the fortress will admit.

"The winds have now continued to blow, for some days, from the south-west, and must have agitated and renewed the air in many of the places on the inhabited face of the rock. The rains, also, that have fallen, have washed the atmosphere of the deepest depressions; but time, the winds that blow from the northward of west, and a lower temperature will be required to exempt from danger a general return to the town, for the present.

"Large fires should be kept up in the most sheltered buildings, for the purpose of producing currents of air. The guard houses, guard beds, and barrack fixtures, should be washed with the watery solution of chloride of lime, after having been whitewashed, fumigated, and thoroughly ventilated.

"As to the cure of those attacked by the disease, I conceive that no treatment can be successful in a malady produced by a poison suspended in the atmosphere, and taken into the blood by respiration, as long as the patient continues to be immersed in the very pool of stagnant air where the poison most abounds. Every treatment, therefore, has failed even in the hands of the most skilful.

"The profuse calomel and bleeding plans, the oil plan, as it is called, have been found almost worse than useless in the crowded situations; whilst the mild harmless plan of lemonade, gentle aperients, diaphoretics, and leeches, has been strikingly successful, where the patients enjoyed free ventilation and large space.

"The measures, unfortunately, but too generally adopted in cases of epidemic, of congregating and insulating the sick in the most limited space possible, and of delaying the release of convalescents, are those that contribute most largely to spread the infection through the affected district, and to increase the mortality of those labouring under the disease, as observed by Baron Dupuytren, in his Report of the Contagion Committee, to the Institute of France, in 1826. Every thing that approaches these depôts of concentrated poison becomes infected, and the greatest part of those who are treated in them perish.

"The Sheds, at present used as a field hospital, on the glacis of the Neutral Ground, are badly situated, both as regards ventilation, (being under the lee of the very highest part of the rock,) and also as regards the diminution of infection. The only winds that can touch them must blow the effluvia of each, in succession, upon those that follow in either direction of the right line in which they are constructed.

"Both the plan and situation of those directed to be raised, according to the views of his Excellency, near the centre of the Neutral Ground, are infinitely superior. They stand *en echelon*, and are thus entirely exempt from the objections made to the others.

"* Yet, notwithstanding these unfavorable circumstances, the sick of the epidemic, hitherto treated at the Sheds, being spread over so much a larger space than those at the Naval Hospital, have recovered with surprising rapidity; nor has there been a single case of infection of any attendant, since I obtained the order for their occupation, and the suspension of all further entry of sick from the Neutral Ground camp into the Naval Hospital.† Should the epidemic, however, continue, and should the convalescents, now increasing fast, be allowed to accumulate, and, with the fresh entries, to crowd the Sheds, they must become a fresh and overflowing source of infection and death.

"The above, sir, is a brief sketch of the first and leading measures which, as occurs to me, ought to be immediately adopted without the loss of a single hour.

"The careful quarantine regulations which you have recommended, render it unnecessary for me to allude to this part of the subject, further than to repeat again that space and ventilation are, in my mind, the best preservatives from infection, as is proved by the disease not having propagated its species, except in a very few

* This paragraph was added to the rough copy, after the original had been sent in to Dr. Broadfoot.—D. B.

† This order was obtained on the evening of the 14th November. See my letter of that date to Dr. Broadfoot, in your November number.—D. B.

doubtful cases, either on the surface of the Bay or the Neutral Ground.

"You will oblige me much by submitting this letter, as early as possible to his Excellency Sir George Don, as I mean that it shall form a part of whatever I may publish on this subject, should his Excellency urge no objection.

"I have the honour to be, sir, your most obedient servant,

(Signed) "DAVID BARRY,
"Physician to the Forces."

"*21st November*, 1828.

"P. S. This letter, intended to have been sent in on the 15th, has been delayed on account of my late indisposition, from which I am now recovering.

"It occurs to me that it would be conferring a most essential and important benefit on those who may happen to succeed us, to leave them a correct matter-of-fact history of the rise and progress of the present epidemic, for their future guidance.

"This could, in my opinion, be best effected by appointing a committee of superior officers, (not medical,) with powers to call before them all persons and papers, &c., on the principle of the committees of the House of Commons.

"Whilst events are fresh in the memory of survivors, whilst there are so many living witnesses still remaining, so many recently written documents still in existence on the spot, the report of this Committee could not fail to be of the very highest interest to science, and to humanity, *I would myself volunteer the duties of secretary*, if thought qualified for that office.* D. B."

Your readers will now, I trust, consider as established,

1st. That, when I wrote the paper, I was a firm believer, as I am now, in the contagious nature or communicability of the Gibraltar fever of 1828.

2d. That I left the investigation of its origin to future inquiry.

3d. That I was the original proposer, at least I thought so at the time, of the commission of inquiry, afterwards appointed by the government at home.

4th. That I volunteered my services to act as secretary to that commission, in the event of its being realized, and therefore had a strong claim to the honour of that very lucrative appointment.

5th. That all these events had already taken place, and were recorded by me and others, in the most official manner,

* "He told me that he had accordingly applied to the Lieutenant Governor, to appoint a commission for the purpose of examining into, and deciding upon the causes of the epidemic." (See M. Chervin's letter to Mr. Fraser, Med. and Phys. Journal, March 1831, p. 207.)—D. B.

eight clear days before the arrival, at the Rock, of my presumed patron Dr. Pym. As a proof, indeed, that I owe nothing to Dr. Pym in the way of promotion, he deemed it necessary, when I was staff surgeon, to place over me, in his list of recommendations, two regimental surgeons, then actually serving under me.

I have been thus particular in laying before you a full account of my very earliest views on the subject of contagion, or rather infection, according to my nomenclature, as applied to the sanitary management of the Gibraltar fever, not alone from their connexion with the two great questions, but also that, from their perfect correspondence with what I have since published, I might, in my future arguments, come before the public "*nitidus in curia*," as the lawyers have it. But I cannot perceive why I should be denied the very ordinary privilege of correcting first impressions, if necessary, by convictions derived from subsequent investigation; nor can I see how "the cause of science" has been so "happily" served by my not having pleaded that privilege, of which, as it now appears, I had no need.

It is really very hard, that, at every step, I should be obliged to trespass on your patience, by stooping to take up and refute some little personal impeachment, to the exclusion of more important matter. But, however I may lament this necessity, it continues to be unavoidable, from the line of argument which it is now but too evident M. Chervin and his followers have resolved to pursue. Ignorance and incompetency, interested and unworthy motives, are to be affixed to all who oppose; but mental acumen, moral weight, independence of principle, and the most disinterested feelings, to those who abet their peculiar dogmas. Discredit is to be cast even on documents which they themselves have deliberately drawn up, and signed, but which they now find may not answer their purpose. It is amusing enough to observe the childish eagerness and ingenuity with which some portions of the same testimony are adopted, and others bitterly rejected, as the schoolboy converts the portrait of grimalkin into the likeness of a rabbit, or vice versâ, by making some slight alterations about the ears and tail.

Mr. Fraser's last letter is made up of special pleadings, and of a style not much to be envied. Finding that he cannot grapple with the facts of the case, the veriest shadows of hearsay are assumed as the grounds of his conclusions. Of this mode of proceeding we have had a pretty fair specimen in the chief accusation, just refuted, as to the history of my opinions. Take another example of manly, straight-forward impeachment, which Mr. Fraser insinuates, in his December letter, to have been of so capital a nature, "that he compelled his fingers to restrain what his pen was going to drop," concerning "the registers of the Civil Hospital having been taken from him, and afterwards withheld, for no other purpose than the perversion of truth." You and your readers have naturally concluded that I was the guilty person whom Mr. F. had in his mind's eye; and, no doubt, he intended to produce that conclusion. But Dr. Broadfoot alone demanded, and had a right, as principal medical officer, to demand and retain Mr. F.'s registers. I defy Mr. Fraser to refute this; and, further, I call upon him to explain his offensive note to its fullest extent.

If any man "loudly asserted, (*and proved*," as interlined by the anomymous hand,) "before the Board, that facts were attempted to be so perverted," nothing can be easier than to specify the occasion and the circumstances.* I, as secretary to the Board, disclaim all recollection of such declaration or proof having been offered; and, at the same time, spurn the foul charge from my own door, with a feeling which I shall not trust myself to express.

Once more, I defy my adversaries to the proof; and let them be assured, that in their silence, or in their reply, public opinion will find the basis of a just decision.

At pages 409 and 411 of your current number, Mr. Fraser has allowed himself to be used as the mouth-piece through which a most serious imputation is uttered to the world, involving, as you will presently perceive, the characters of more than one medical officer, besides the hospital discipline

* The note, and the letter to which it was appended, were both in the handwriting of Mr. Fraser, but interlined by another hand, in the manner described.

Mr. F., in his letter of the 15th January, explanatory of the offensive interlineations, calls the whole "an exact copy, though not in the same handwriting throughout, of one which he had made out, and forwarded from *this!*" (Chatham) to London. It remains, however, still to be explained,

1st. Why Mr. Fraser should have employed an anonymous writer to add three different editions of interlineations, (in some places wafered on, over each other,) to a letter copied by himself from an original, which we are led to presume was also penned by himself, "and sent up" from "this!" i. e. Chatham, to London?

2dly. How a letter, so interlined, can be said to be an exact copy of any original?

3d. Why the original itself was not sent to the press, particularly as it had been "sent up" before the copy in which Mr. F. omitted the offensive interlineations, the originals of which must, according to the explanatory letter, have been contained in the text of the letter first "sent up," and penned, as we must believe, by Mr. Fraser?—D. B.

of a distinguished corps.. In referring to the little statistic abstract of admissions and deaths of the sick of the 12th foot, in the epidemic division of the camp hospital, given by me in your November number, he more than insinuates that the returns, of which that abstract is composed, were fabricated for the purposes of "deception." But Mr. F., when he permitted such a charge to be published in his name, and Mr. Amiel, when he lent, if indeed he did lend, his name to such a publication, must have forgotten that no less than *thirty-one* "cases of fever" are individually and numerically returned by Mr. Amiel himself and his assistant surgeon, as having been treated in that very Sheds hospital, and within the time specified by me.

But, to place this matter beyond the pale of controversy for ever, the Gibraltar quarterly returns of the sick, signed by the principal medical officer, (now lying at the Board in Berkeley street,) for the military quarters ending 20th September and 20th December, 1828, give for the 12th regiment, "admitted," under the head of "Febris epidemica," 276.

The nominal "return of fever cases" of the 12th regiment, admitted into the Naval Hospital from 1st September to 20th December, 1828, signed R. Amiel, surgeon, gives

| | |
|---|---|
| Individual names . . . | 220 |
| Relapses . . . . . . . | 26 |
| | 246 |

The nominal return of "fever cases" admitted into the Sheds hospital during the same period, (the first admission being on the 15th November, the day of its establishment, the last but one on the 14th December,* gives

| | |
|---|---|
| Individual names . . . | 31 |
| | 277 |

Observe, that both the above nominal returns were given in by Mr. Amiel to the principal medical officer at Gibraltar, sometime after the 20th of December, the day to which they are both made up.

My statement of twenty-two admissions of the 12th regiment into the Sheds was founded on a nominal return made out on the spot, and furnished to me by Assistant-surgeon

* Only four cases were admitted in December, and one of these is noted in the Sheds return, given in by Mr. Amiel, as a relapse.—D. B.

Dick, who was in personal charge of them at the time. From what I know of that most efficient and zealous officer, I can vouch for his correctness. I have no doubt, however, that Mr. Amiel, for whom I have hitherto entertained the highest respect, will, with the assistance of Mr. Fraser and his friends, be able to explain the excess of nine cases of "febris epidemica" in his returns, over mine for the Sheds hospital; and also to reconcile this excess with the declaration said to have been authorised by him, "that very few of them (viz. the twenty-two), indeed, ought to have been at all classed under *that head*." The *onus probandi* is certainly with these gentlemen for the present.

Of the declaration by Mr. Gallice, I shall merely remark, that it forms perhaps the only instance upon record, where the conversation of a guest, at a mess-table, has ever been published by a British officer, himself one of the hosts, particularly with motives such as those of Mr. G. appear to have been. Few, I should think, will envy him this unique species of notoriety.

With the most unfeigned regard, nay, with strong feelings of personal friendship for that truly excellent officer and good man, the late surgeon of the 42d regiment, I cannot comprehend how those who have quoted his declaration, can make *one* "unfortunately a considerable proportion" of *eleven*.

When Mr. Fraser's observations on the medical history of the 43d regiment, with which he has latterly become so very intimate, shall have been duly authorized, or when these observations shall have appeared in a substantive, affiliated form, they may perhaps meet with that notice from me, to which, in their present unauthenticated shape, they are not legitimately entitled. In the mean time, one word as to mercury.

Aggravated yellow fever bears a stronger resemblance to acute scorbutus, or sea scurvy, than to any other disease; witness, the soft and bleeding gums, livid, cutaneous blotches, decomposition of the blood, speedy dissolution in both. Of the treatment of this latter disease, Sir G. Blane, in his "Medical Logic," p. 272, gives the following anecdote, on the authority of Dr. Kramer:

"That some of the medical officers of the imperial armies in Hungary did, in the year 1720, subject to a mercurial course four hundred men ill of the sea scurvy, every one of whom died."

Here I take leave of Mr. Fraser's rambling letters, for the present; and shall now proceed to the facts more immediately connected with the two leading questions proposed at the commencement of this communication.

"La science en general se compose de faits partiels bien constatés."

*London; May 5th,* 1831.

(To be continued.)

